# Atrial septal defect becoming clinically evident after cardiac surgery for annuloaortic ectasia: a case report

**DOI:** 10.1093/ehjcr/ytae299

**Published:** 2024-06-18

**Authors:** Kentaro Meguro, Toshimi Koitabashi, Teppei Fujita, Junya Ako

**Affiliations:** Department of Cardiovascular Medicine, Kitasato University, 1-15-1 Kitasato, Minami-ku, Sagamihara, Kanagawa 252-0374, Japan; Department of Cardiovascular Medicine, Kitasato University, 1-15-1 Kitasato, Minami-ku, Sagamihara, Kanagawa 252-0374, Japan; Department of Cardiovascular Medicine, Kitasato University, 1-15-1 Kitasato, Minami-ku, Sagamihara, Kanagawa 252-0374, Japan; Department of Cardiovascular Medicine, Kitasato University, 1-15-1 Kitasato, Minami-ku, Sagamihara, Kanagawa 252-0374, Japan

**Keywords:** Atrial septal defect, Aortic root dilatation, Right ventricular overload, Annuloaortic ectasia, Case report

## Abstract

**Background:**

Right ventricular volume overload is the key finding in a patient with previously undiagnosed atrial septal defect (ASD).

**Case summary:**

A 68-year-old female was referred to our hospital due to progressive pulmonary artery dilatation observed on her chest X-ray. Echocardiography revealed a secundum ASD with right ventricular dilatation. She had undergone aortic root replacement and aortic valve replacement for annuloaortic ectasia and aortic insufficiency 12 years prior to the diagnosis. She was also diagnosed with Marfan syndrome, which was supported by family histories. Computed tomography did not show a secundum ASD before the surgery. We finally closed the secundum ASD with catheter closure device.

**Discussion:**

Ascending aneurysm might mask the presence of secundum ASD. Monitoring the change in pulmonary artery dilatation overtime is useful for the diagnosing secundum ASD.

**Learning points**

- Ascending aneurysm may mask the presence of atrial septal defect.
- The key finding in a patient with previously undiagnosed atrial septal defect is right ventricular overload.

## Introduction

Atrial septal defect (ASD) is one of the most common congenital heart diseases in adults.^1^ Surgical indications for ASD closure include right ventricle volume overload, pulmonary to systemic flow ratio (Qp:Qs) > 1.5, and suspicion of paradoxical embolism.^2^ Right ventricular volume overload is the key finding and best characterizes the haemodynamic relevance of the ASD in a patient with previously undiagnosed ASD. However, in patients with annuloaortic ectasia (AAE), ASD can be easily overlooked by its anatomical proximity to the dilated aortic root. We report a case of secundum ASD with right ventricular volume overload becoming evident 12 years after aortic replacement for AAE.

## Summary figure

**Table:** 

Background	Marfan syndrome with family history
12 years prior to the ASD diagnosis	Aortic root replacement and aortic valve replacement with mechanical valve for AAE and aortic insufficiency
ASD diagnosis	Pulmonary artery dilatation and ASD diagnosis by echocardiography
2 years after the diagnosis	Catheter closure using 37 mm GORE CARDIOFORM ASD Occluder

## Case presentation

**Figure 1:**
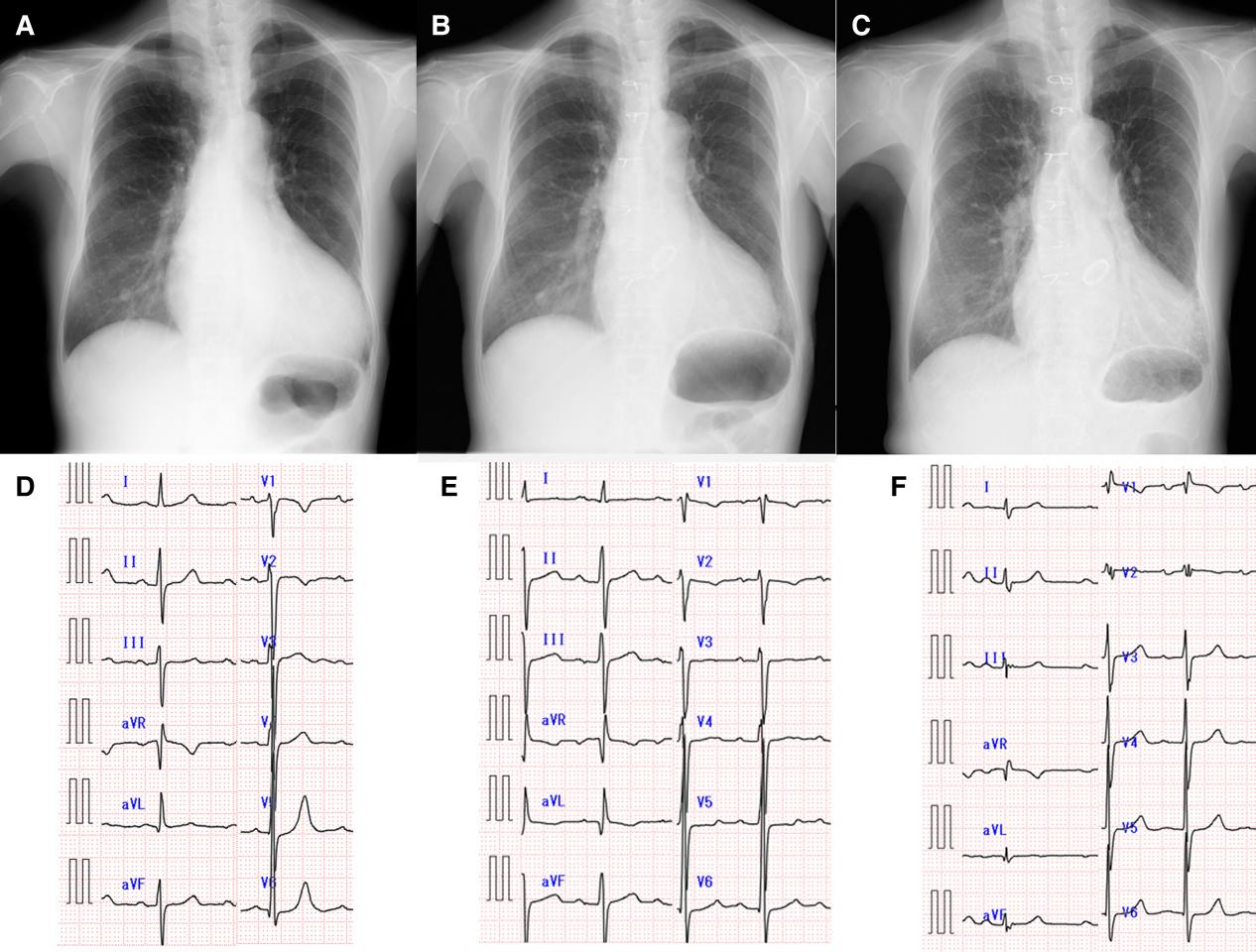
Progressive pulmonary artery dilatation and electrocardiogram (ECG) change after the surgery of aortic root replacement and aortic valve replacement. (*A* and *D*) Chest X-ray and ECG before the surgery. (*B* and *E*) Chest X-ray and ECG after the surgery. (*C* and *F*) Chest X-ray and ECG at the time of atrial septal defect diagnosis.

A 68-year-old female with a history of aortic root replacement and aortic valve replacement with mechanical valve for AAE and aortic insufficiency 12 years prior was referred to our hospital due to pulmonary artery dilatation. She was diagnosed with Marfan syndrome. Her son and grandson were also diagnosed with Marfan syndrome. During the annual follow-up after the surgery, her chest X-ray showed progressive pulmonary artery dilatation (*Figure 1A–C*), although her functional class remained New York Heart Association I. She took warfarin 1.5 mg, aspirin 100 mg, carvedilol 10 mg, enalapril 1.25 mg, and omeprazole 10 mg daily. Her blood pressure was 126/73 mmHg with a regular pulse rate of 85/min. Her lungs were clear, and she had no signs of heart failure. The sound of the mechanical valve was heard during auscultation without any significant murmur. Laboratory findings showed anaemia (haemoglobin 10.0 g/dL), normal renal function (estimated glomerular filtration rate 61 mL/min/1.73 m^2^), and a slight elevation of N-terminal pro-brain natriuretic peptide to 8.0 pmol/L. Her electrocardiogram (ECG) showed new incomplete right bundle branch abnormality (iRBBB) after the surgery (*Figure 1D–F*).

**Figure 2:**
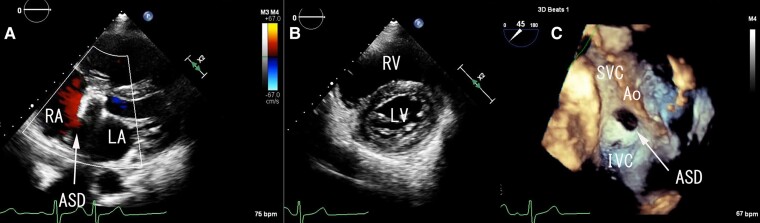
Atrial septal defect diagnosis in echocardiogram. (*A*) Secundum atrial septal defect and left-to-right shunt in transthoracic echocardiogram. (*B*) Short-axis view in transthoracic echocardiogram during the diastolic phase. It is considered that there was right ventricular volume overload due to the left ventricle being compressed during the diastolic phase. (*C*) Crescent-shaped secundum atrial septal defect with aortic and superior rim deficiency in transoesophageal echocardiogram. Videos of (*A*)–(*C*) are also provided in Supplementary material online, *Video S1A–C*. RA, right atrium; LA, left atrium; ASD, atrium septal defect; RV, right ventricle; LV, left ventricle; Ao, aorta.

Her transthoracic echocardiogram revealed the 10 mm size of secundum ASD and the left-to-right shunt with right ventricle enlargement (*Figure 2A* and *B*). Her estimated pulmonary artery pressure (PAP) was 37.2 mmHg in her transthoracic echocardiogram. Despite the annual follow-up echocardiogram, secundum ASD was not diagnosed at the other hospital. Her transoesophageal echocardiogram (TOE) showed the 7 × 18 mm crescent-shaped secundum ASD with aortic and superior rim deficiency (*Figure 2C*). The presence of ASD had not been confirmed at the time of aortic root replacement despite the use of TOE and cannulation from the right atrial appendage during the surgery. Her cardiac catheterization showed high Qp:Qs of 1.74 and normal pulmonary resistance (1.9 WU); therefore, we considered that her ASD closure is recommended regardless of the symptoms.^2^

**Figure 3:**
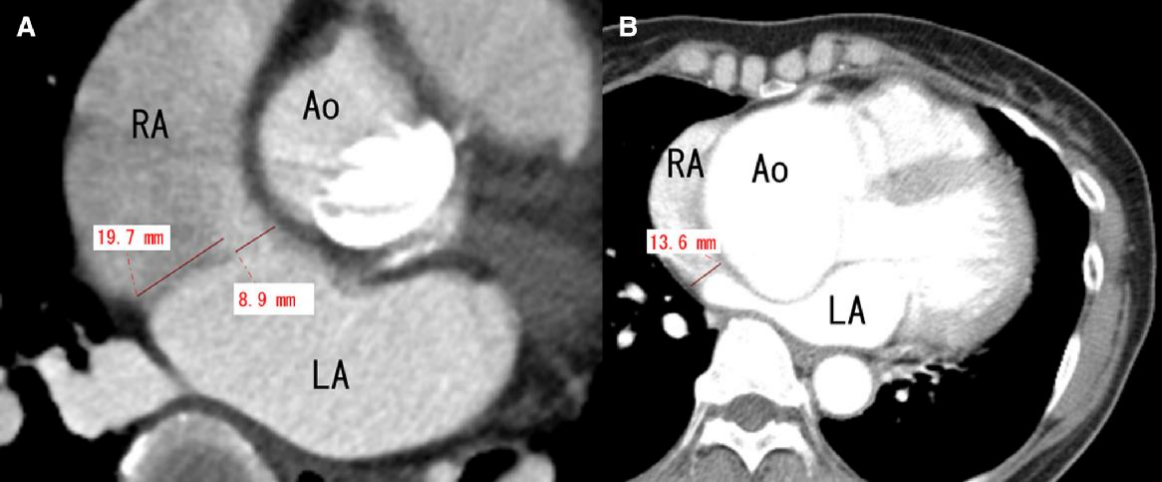
Computed tomography image. (*A*) At the time of secundum atrium septal defect diagnosis. (*B*) Before the aortic root replacement and aortic valve replacement (12 years before the atrium septal defect diagnosis).

Computed tomography (CT) was also performed to exclude the coexistence of other cardiac and vascular abnormalities. Her CT showed the secundum ASD with a size of 8.9 mm and 19.7 mm long posterior rim (*Figure 3A*). Her CT before the surgery of aortic root replacement did not show any defect between the right and left atrium nor right ventricle dilatation (*Figure 3B*). The length of atrial septum, which was compressed by the ascending aneurysm, was 13.6 mm. Since the distance of atrial septum before surgery was shorter than the length of posterior rim in CT, we considered that the secundum ASD shunt flow did not exist at the time of surgery, which made it very difficult to diagnose its presence. The new iRBBB after the surgery also supports this consideration.

**Figure 4:**
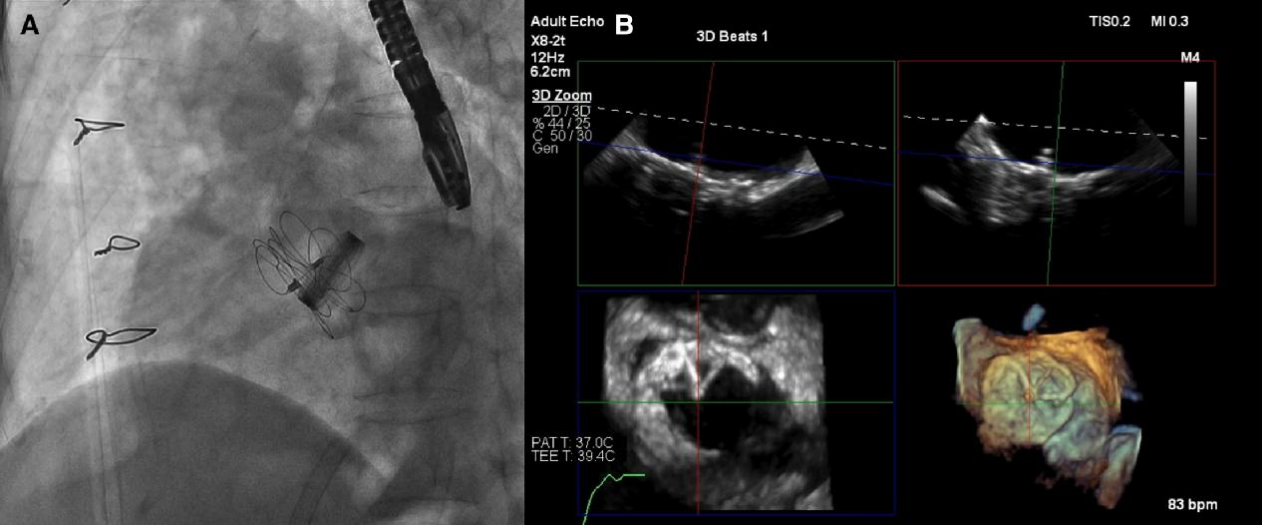
Closure with 37 mm GORE CARDIOFORM ASD Occluder device. Videos of (*A*) and (*B*) are also provided in Supplementary material online, *Video S2A* and *B*. (*A*) Angiogram. (*B*) Transoesophageal echocardiogram.

The decision was made to perform percutaneous secundum ASD closure with GORE CARDIOFORM ASD Occluder (GCA, W.L. GORE & Associates, Flagstaff, AZ). We finally closed her secundum ASD with 37 mm GCA (*Figure 4*) with the guidance of TOE. She was discharged from our hospital 2 days after the procedure. Her echocardiogram 5 months post-procedure revealed a decrease in estimated PAP to 27.2 mmHg and an improvement in the right ventricular volume overload.

## Discussion

We describe a case of secundum ASD becoming clinically evident after aortic replacement in a patient with AAE. Her chest X-ray showed progressive pulmonary artery dilatation after cardiac surgery. Right ventricular volume overload was observed, and we finally diagnosed ASD by transthoracic echocardiogram.

At the time of aortic replacement, the right ventricular overload and secundum ASD were not clearly observed. We considered two possible explanations for this.

1. This patient had had secundum ASD before progression of AAE; however, the secundum ASD was covered by the huge AAE because the distance between posterior wall and aorta became less than the length of septum secundum.
2. Patent foramen ovale had been present in this patient, and the shunt between the right and left atrium became apparent after the cardiac surgery. Huge AAE might have caused the deformation of atrial structure, especially deviation towards the left atrium and elongation of the edge of septum secundum. The cardiac surgery of AAE corrected the size and the position of ascending aorta (*Figure 3*), and the defect of atrial septum could become evident.

It is reported that an aortic aneurysm or elongation might cause a deformity of the atrial septum and blood flow between the right and left atrium in patients with platypnoea–orthodeoxia syndrome (POS),^3,4^ which is characterized by objective evidence of hypoxaemia in the upright position that resolves in the supine position.^5^ In this patient, hypoxia in upright position was not observed; however, the mechanism of emergence of the blood flow between the right and left atrium could be similar to that of POS.

The Marfan syndrome is associated with tissue fragility, leading to the progressive aortic dilatation and dissection.^6^ It is reported that Marfan syndrome also causes pulmonary artery dilatation^7^; however, the change was only detected by magnetic resonance imaging. Therefore, we believe that the dilatation of the pulmonary artery is mainly due to secundum ASD. Amplatzer Septal Occluder (ASO, Abbott Medical, Abbott Park, IL) is the most commonly used device for secundum ASD closure and can cause rare but potentially serious adverse event due to erosion. It is reported that aortic and superior rim deficiency increased the risk of device erosion.^8^ The incidence of erosion after ASO device closure is estimated as 1–3 in every 1000 cases, and dilatation of aortic root is also reported to be the risk of erosion.^9^ On the other hand, there have been no device erosions reported with GCA. In this case, TOE showed aortic and superior rim deficiency (*Figure 2C*). Since she also underwent the aortic root replacement due to AAE, we considered that her anatomy was high risk for the device erosion with ASO, and GCA was the best treatment option.

## Conclusion

Secundum ASD was diagnosed for the first time 12 years after aortic replacement for the AAE. The dilated aortic root may have obstructed the shunt flow, possibly masking the presence of secundum ASD. Device closure for secundum ASD was feasible in patients who had undergone aortic root replacement.

## Supplementary Material

ytae299_Supplementary_Data

## Data Availability

The data underlying this article are available in the article and in its online Supplementary material.

## Lead author biography

Kentaro Meguro is a cardiac intervention specialist who works for Kitasato University Hospital in Japan. He received his medical degree at the Hokkaido University in 1999. He obtained a Doctor of Philosophy in Medicine from the University of Tokyo. He worked and learned structural heart interventions in Henri Mondor Hospital at Creteil, Paris, in France in 2010–11.

## Supplementary material

**Consent:** The authors confirm that written consent for submission and publication of this case report, including the accompanying images and associated text, has been obtained from the patient in line with the COPE guidelines.

**Funding:** None declared.
